# Effects of adaptation time and inclusion level of sugar beet pulp on nutrient digestibility and evaluation of ileal amino acid digestibility in pigs

**DOI:** 10.5713/ajas.18.0181

**Published:** 2018-08-27

**Authors:** Ze Yu Zhang, Shuai Zhang, Chang Hua Lai, Jin Biao Zhao, Jian Jun Zang, Cheng Fei Huang

**Affiliations:** 1 State Key Laboratory of Animal Nutrition, College of Animal Science and Technology, China Agricultural University, Beijing 100193, China

**Keywords:** Adaptation Time, Amino Acid, Digestibility, Inclusion Level, Pig, Sugar Beet Pulp

## Abstract

**Objective:**

Two experiments were conducted to determine the effects of adaptation time and inclusion level of sugar beet pulp (SBP) on nutrient digestibility and to evaluate the ileal amino acid digestibility of SBP fed to pigs.

**Methods:**

In Exp. 1, thirty-six crossbred barrows (85.0±2.1 kg) were allotted to 6 diets in a completely randomized design with six replicates per diet. Diets included a corn-soybean meal diet and 5 test diets containing 14.6%, 24.4%, 34.2%, 43.9%, or 53.7% SBP, respectively. The adaptation time consisted 7, 14, 21, or 28 d consecutively for each pig followed by 5 d for fecal collection. Feces were collected from d 8 to 13, d 15 to 20, d 22 to 27, and d 29 to 34, respectively. In Exp. 2, six pigs (35.1±1.7 kg) with T-cannulas at the terminal ileum were fed to 3 diets in a replicated 3×3 Latin square design with 3 periods and 2 replicate pigs per diet. Each period consisted 5 d for diet adaptation followed by 2 d for digesta collection.

**Results:**

The digestible energy (DE) value and the apparent total tract digestibility (ATTD) of gross energy (GE), dry matter (DM), ash, and organic matter in diets linearly decreased (p< 0.05) as the adaptation time increased or as the dietary SBP increased, while the ATTD of neutral detergent fibre and acid detergent fibre in diets linearly increased (p<0.01) as the dietary SBP increased. The DE value and the ATTD of GE and crude protein (CP) in SBP linearly increased (p<0.05) as the adaptation time increased, while the ATTD of CP in SBP linearly decreased (p<0.01) as the inclusion level increased. The standardized ileal digestibility of Lys, Met, Thr, and Trp in SBP was 37.03%, 51.62%, 40.68%, and 46.22%, respectively.

**Conclusion:**

The results of this study indicated that the ATTD of energy and nutrients were decreased as inclusion rate of SBP increased.

## INTRODUCTION

Monogastric animals cannot secret endogenous enzymes into the small intestine to degrade dietary fiber, but the dietary fiber can be fermented in the hindgut of these animals [1]. Volatile fatty acids generated from fiber fermentation in the hindgut can provide 15% to 30% of the net energy requirements for maintenance in pigs [2–4]. However, diets with a high fiber content usually have low apparent total tract digestibility (ATTD) and apparent ileal digestibility (AID) of nutrients [5–7]. The magnitude of the negative effects of fiber on the nutrient digestibility may be affected by the inclusion level of dietary fiber [6]. Moreover, it usually takes some time for pigs and the microbiota in the hindgut to adapt to high fiber diets [6,8,9]. A previous study on pregnant sows reported that 10 days of adaptation to a diet with a high level fermentable non-starch polysaccharides (30%) is needed [10]. Another study found that the gut microbiota in pigs need at least 3 weeks and up to 6 weeks to adapt to a new diet [8]. However, these studies were conducted with only one dietary fiber level. It is still not clear whether there is an interaction effect between the adaptation time of diets and the dietary fiber level. Sugar beet pulp (SBP) has comparable of gross energy (GE) and crude protein (CP) contents to corn grains [11]. The high fiber contents in SBP limit its utilization as an energy or protein source in swines’ diet. In addition, little information is available on the nutritional values and digestibility of nutrients and amino acids (AA) of SBP in growing pigs. Therefore, the objectives of this study were: i) to test the hypothesis that there was an interaction effect of adaptation time and inclusion level of SBP on nutrient digestibility in pigs, and ii) to determine the ileal digestibility of CP and AA in SBP fed to growing pigs.

## MATERIALS AND METHODS

The protocol for all animal procedures was approved by the Institutional Animal Care and Use Committee at China Agricultural University (Beijing, China).

### Experiment 1: Effect of adaptation time and inclusion level of sugar beet pulp on energy value and nutrient digestibility in growing pigs

#### Experimental design and diets

Thirty-six crossbred barrows (Duroc×Landrace×Large White) with an average of 85.0±2.1 kg body weight (BW) were allotted to a completely randomized design with 4 adaptation time and 6 diets. The adaptation time consisted 7, 14, 21, or 28 d consecutively for each pig, with feces collected from d 8 to 13, 15 to 20, 22 to 27, and 29 to 34, respectively. The experimental diets included a corn-soybean meal-based diet containing 76.6% corn, 21.0% soybean meal (SBM), 1.0% limestone, 0.6% dicalcium phosphate, 0.3% salt, and 0.5% vitamin-mineral premix. The 5 test diets were formulated by substituting the energy source of corn and SBM in the basal diet with 15%, 25%, 35%, 45%, or 55% of SBP. The concentration of SBP was 14.6%, 24.4%, 34.2%, 43.9%, and 53.7% in the five complete test diets, respectively. Vitamins and minerals were supplemented in all diets to meet or exceed the nutrient requirements for 75 to 100 kg barrows [11]. The GE and analyzed proximate compositions of corn and SBP were presented in Table 1. The ingredient and analyzed compositions of six experimental diets are presented in Table 2.

#### Animal housing, feeding, and sample collection

All pigs were individually housed in stainless-steel metabolism crates (1.4×0.9×0.7 m^3^) at the Fengning Swine Research Unit of China Agricultural University (Chengde, China). The crates had adjustable sides and were in a room with temperature and light controlled at 22°C±2.5°C and 12 h of light and 12 h of dark, respectively. Humidity varied from 55% to 65% during the experiment. An adjustable screen was placed under each crate for total feces collection. Pigs were acclimated to the new environment for 7 d and fed a commercial corn-SBM-based diet before the beginning of the experiment. Experimental diets were daily offered to pigs at a level of 3% of their initial BW and provided in 2 equal meals at 0830 h and 1630 h. Feed refusals were collected and weighed daily. Pigs were fed the same experimental diets throughout the whole experimental period. The adaptation time lasted for 7 days and then feces were quantitatively collected for 5 days during each collection period. Feces were consecutively collected from d 8 to 13, d 15 to 20, d 22 to 27, and d 29 to 34, respectively. Fresh feces were stored at −20°C immediately after collected. At the end of each collection period, the collected feces were weighed and thawed, and then homogenized thoroughly within pig and collection period. A sub-sample of feces from each pig in each collection period was dried in a forced-air oven at 65°C for 72 h. Dried feces were ground through a 1-mm screen before chemical analysis. All pigs had *ad libitum* access to water via a drinking nipple.

#### Chemical analysis

Samples were analyzed for GE via an adiabatic oxygen bomb calorimeter (Parr 6300 Calorimeter; Parr Instrument Company, Moline, IL, USA). Other analysis including DM, CP, and ash analyzed according to AOAC [12]. The ether extract (EE) was analyzed by a previous method [13]. Total dietary fiber (TDF) and insoluble dietary fiber (IDF) in SBP or corn grain were also determined according to AOAC [12]. The concentration of soluble dietary fiber (SDF) was calculated as the difference between TDF and IDF. Neutral detergent fiber (NDF) and acid detergent fiber (ADF) were determined using filter bags and fiber analyzer equipment (Fiber Analyzer; ANKOM Technology, Macedon, NY, USA) following a modification of the procedures [14]. The concentration of NDF was analyzed using heat-stable α-amylase and sodium sulfite without correction for insoluble ash. Organic matter (OM) was calculated as the difference between DM and ash. All chemical analyses were conducted in duplicates.

#### Calculations

The digestibility of GE and other nutrients in feed and SBP were calculated according to the difference method [15], and the DE and metabolizable energy were calculated by multiplying the GE content and the digestibility of GE in feed or SBP, respectively.

### Experiment 2: Amino acid digestibility in sugar beet pulp

#### Experimental design and diets

Six pigs (35.1±1.7 kg BW) fitted with T-cannulas at the terminal ileum according to the method [16] were fed to 3 diets in replicated 3×3 Latin square design with 3 periods and 2 replicate pigs per diet in each period. The 3 experimental diets included a corn diet containing 96.6% corn, a SBP diet containing 50% corn starch, 30% SBP, 15% sucrose, and 2% soybean oil, and a nitrogen-free (N-free) diet containing 73.35% corn starch, 15% sucrose, 4% acetate cellulose, and 3% soybean oil. The N-free diet was used to determine the basal ileal endogenous N losses. Chromic oxide, as an indigestible index, was included in all diets (0.3%) for calculating the ileal digestibility of AA. Vitamins and minerals were supplemented in all diets to meet or exceed the estimated nutrient requirements for growing pigs [11]. The analyzed AA contents of SBP are presented in Table 1. Ingredients, analyzed CP, and AA compositions of the 3 experimental diets are presented in Table 3.

#### Animal housing, feeding, and sample collection

All pigs were individually housed in stainless-steel metabolism crates (1.4× 0.9×0.7 m^3^) at the metabolism room located at China Agricultural University. After 10 d of recovery from the T-cannulas surgery at the terminal ileum, pigs were fed experimental diets. Each period consisted 5 d for diet adaptation followed by 2 d for digesta collection.

Experimental diets were daily offered to pigs at a level of 4% of their initial BW and provided in 2 equal meals at 0730 h and 1630 h each day. The digesta collection lasted for 9 h daily beginning at 0800 h according to the procedures described by Stein et al [16]. On d 6 and 7, plastic bags were attached to the barrel of the cannulas and removed whenever they were filled with digesta and then stored at −20°C. At the end of the experiment, digesta samples were thawed, mixed by pig and period, subsampled, and lyophilized in a vacuum-freeze dryer (Tofflon Freezing Drying Systems, Minhang District, Shanghai, China). All pigs had *ad libitum* access to water via a drinking nipple.

#### Chemical analysis

Before analysis, SBP, diets, and digesta were ground through a 1-mm screen and mixed thoroughly. Analyses of the AA content in all samples were conducted according to Li et al [17]. For all AA, excluding Met, Cys, and Trp, samples were hydrolyzed with 6 *N* HCl at 110°C for 24 h and then analyzed using an Amino Acid Analyzer (Hitachi L-8900; Hitachi Ltd., Tokyo, Japan). The sulfur AA (Met and Cys) were subjected to cold performic acid oxidation overnight and hydrolyzed with 7.5 *N* HCl at 110°C for 24 h before measured using an Amino Acid Analyzer (Hitachi L-8900, Hitachi Ltd., Japan). Estimate of Trp was made by hydrolyzing the sample with LiOH for 22 h at a constant temperature of 110°C and then analyzed using High Performance Liquid Chromatography (Agilent 1200 Series; Agilent Technologies Inc., Santa Clara, CA, USA). Analysis of Cr in the diets and digesta was conducted using a polarized Zeeman Atomic Absorption Spectrometer (Hitachi Z2000; Hitachi Ltd., Japan) after nitric acid–perchloric acid wet ash sample preparation. All analyses were conducted in duplicate.

#### Calculations

The AID and SID of AA was calculated using the following equation [18]:

$$\text{AID}=[1-({\text{AA}}_{\text{d}}/{\text{AA}}_{\text{f}})\times ({\text{Cr}}_{\text{f}}/{\text{Cr}}_{\text{d}})]\times 100\%,$$

where AA_d_ and Cr_d_ are the concentrations of AA and Cr in the ileal digesta (g/kg of DM) and AA_f_ and Cr_f_ are the concentrations of AA and Cr in the test diets (g/kg of DM), respectively. The AID of CP was calculated using the same equation.

The endogenous loss of N for each AA was measured from pigs fed the N-free diet based on the following equation:

$${\text{IAA}}_{\text{end}}=[{\text{AA}}_{\text{d}}\times ({\text{Cr}}_{\text{f}}/{\text{Cr}}_{\text{d}})],$$

where IAA_end_ is the basal endogenous loss of an AA (g/kg of DMI), and AA_d_ and Cr_d_ represent the concentrations of AA and Cr in the ileal digesta of the pigs fed the N-free diet, respectively. The concentration of Cr in the N-free diet is represented by Cr_f_. The basal endogenous loss of CP was determined using the same equation.

The average IAA _end_ of the 6 pigs fed the N-free diet was used to calculate the SID of AA in all diets. Standardized ileal digestibility was then calculated using the following equation:

$$\text{SID}=[\text{AID}+({\text{IAA}}_{\text{end}}/{\text{AA}}_{\text{f}})\times 100\%].$$

### Statistical analysis

Data were checked for normality and outliers were identified using the UNIVARIATE procedure of SAS 9.4 (SAS Institute, Cary, NC, USA). An observation was considered as an outlier if the value was more than 3 standard deviations away from the grand mean. In Exp.1, data were analyzed using the MIXED procedure of SAS (SAS Inst. Inc., USA) in two-way analysis of variance. The statistical model included the main effects of inclusion level and adaptation time and their interaction effect. Repeated measurements were considered when analyzing the effects related to adaptation time. Pig was treated as the experimental unit. Treatment means were separated using the LSMEANS statement. Orthogonal polynomial contrasts were conducted to determine linear and quadratic effects of inclusion level or adaptation duration. In Exp. 2, data were analyzed using the MIXED procedure of SAS, with a pig as the experimental unit. The statistical model included the fixed effect of diet, and the random effects of period and animal. Treatment means were separated using the LSMEANS statement, and Student t test was used to compare the differences in AA digestibility between corn and SBP. In both experiments, statistical significance and tendency were considered at p<0.05 and 0.05≤p<0.10, respectively.

## RESULTS

### Chemical composition of sugar beet pulp and corn grain

Analyzed composition of SBP used in Exp. 1 and 2, and the corn grain used in Exp. 2 are presented in Table 1.

### Effects of adaptation time and inclusion level of sugar beet pulp on the apparent total tract digestibility of nutrients and digestible energy value in diet

No interactive effects between adaptation time and inclusion level of SBP on the DE value and the ATTD of GE, CP, DM, ash, OM, NDF, and ADF in diets were observed (Table 4). The concentration of DE and the ATTD of GE, DM, ash, and OM in diets linearly decreased (p<0.05) as the adaptation time increased, regardless of the inclusion level of SBP. The ATTD of DM, ash, OM, and NDF quadratically changed as the adaptation time prolonged from 7 to 28 d: decreased from 7 to 21 d and then increased from 21 to 28 d (p<0.05). The DE value of diet varied from 3,295 to 3,259 kcal/kg (as-fed basis) when the adaptation time increased from 7 to 28 d.

The concentration of DE and the ATTD of GE, CP, DM, ash, and OM linearly decreased (p<0.01) as the inclusion level of SBP increased from 0% to 53.7%, regardless of the adaptation time. The ATTD of NDF and ADF linearly increased (p< 0.01) as the inclusion level of SBP increased. The concentration of DE varied from 3,529 to 3,075 kcal/kg (as-fed basis) as the inclusion level of SBP increased from 0% to 53.7%.

### Effects of adaptation time and inclusion level on the digestible energy value and the apparent total tract digestibility of gross energy and crude protein in sugar beet pulp

An interactive effect (p<0.05) on the DE value and the ATTD of GE and CP in SBP were observed between the adaptation time and inclusion level (Table 5). The concentration of DE and the ATTD of GE and CP in SBP linearly increased (p<0.05) as the adaptation time prolonged from 7 to 28 d. The DE value and the ATTD of GE in SBP varied from 2,869 to 3,061 kcal/kg (as-fed basis) and 74.08% to 78.98%, respectively. The ATTD of CP in SBP varied from 64.92% to 79.16%.

### Ileal amino acid digestibility of sugar beet pulp

The AID and SID of CP and all detected AA in SBP were less (p<0.05) than those in corn grain (Table 6).

## DISCUSSION

Sugar beet pulp sample used in the current experiment contained lower CP, EE, and TDF values but greater ash content compared with the previous reports [6,11,19]. Factors such as the variety of the beet and processing procedure of the SBP can affect the composition of the SBP. The ratio of SDF to TDF for the SBP sample used in this study was 40.7% and which was greater than that of 32.0% reported by Zhang et al [19]. This indicated that SBP is an ingredient with high soluble fiber content. It is well known that SDF can be fermented rapidly by the bacteria in the hindgut of pigs to produce short-chain fatty acids [20,21]. Therefore, sugar beet pulp was included into the diets fed to sows and growing pigs as a source of soluble dietary fiber.

### Effects of inclusion level of sugar beet pulp on the digestible energy value and the apparent total tract digestibility of nutrients in diets

The decreased concentrations of DE value and ATTD of GE in diets as dietary SBP level increased from 0% to 53.7% agreed with the previous results [19]. Another study showed that the ATTD of GE decreased when the dietary fiber level increased from 12.0% to 38.0%, in which the fibers are mixture of wheat bran, maize bran, soybean hulls, and SBP [22]. The reduction in energy digestibility with increased dietary fiber level can be attributed to the increased cell wall components from the fiber sources which are less digestible [1]. In addition, high fiber ingredients used in diets can dilute the energy fraction of the feed and then decrease the digestibility of energy and the other nutrients [7,9]. Another possible reason may be that the increased digesta viscosity induced by SBP impeded the diffusion rate of nutrients from the lumen to the epithelial cells, which caused a reduction in the absorption of the nutrients [23].

The decreased ATTD of CP, DM, ash, and OM with in creased dietary SBP level in our research agreed with Bindelle et al [20], who reported that the ATTD of CP, DM, ash, and OM linearly decreased when a corn-SBM-based diet supplemented with SBP at levels of 0%, 10%, 20%, and 30% was fed to growing pigs. It has been reported in rats that a 15% to 30% of fiber composition in SBP presents as a form of pectin [24], which can increase digesta viscosity and thus inhibit enzyme activity and possibly increase proteolytic enzyme secretion [25]. The decreased ATTD of CP in diets may be due to the negative effect of NDF level on N digestibility [26]. In addition, increased digesta viscosity and then decreased proteolytic enzymatic digestion of CP were induced by high fiber from SBP [27–29]. Another possible reason for the decreased ATTD of CP in diets with increased SBP may be due to higher endogenous losses of N because of increased secretion of endogenous amino acids induced by high fiber [20].

Increased ATTD of NDF with increased dietary SBP agreed with the report that the ATTD of NDF linearly increased when dietary SBP increased from 0% to 30% [20] or when dietary wheat bran increased from 9.7% to 48.3% [30]. Zhang et al [19] found that the ATTD of TDF, SDF, and IDF increased as the dietary SBP increased from 0% to 55.0%. This may be due to the increased viscosity of digesta induced by increasing the levels of SBP, which can prolong the digesta passage rate and leave more time for fermentation of fiber in the lower gut by bacteria [31]. Therefore, the ATTD of nutrients in feed is affected by the source and level of fiber.

### Effect of adaptation time on the digestible energy value and the apparent total tract digestibility of components in diets

Dietary fiber is mainly fermented in the hindgut of monogastric animals. It has been reported that sows and adult pigs had greater digestibility of fiber than young pigs. The main reasons may be that adult pigs have a larger, more developed intestinal tract and thus, greater intestinal volume which allows for more exposure of feed to enzymes and bacteria and more absorption of nutrients in the small and large intestine [32–34]. For this reason pigs with 85.0 kg BW were chosen in Exp. 1. However, Kass et al [35] found that digestive capacity was negatively related to the live weight of pigs. The different dietary fiber type of SDF and IDF can be a plausible explanation for the different results among different experiments. Previous studies showed that SDF are more likely to produce modifications in the physico-chemical properties of colonic digesta without changing the colonic microbiota diversity in the proximal colon of monogastric animals than IDF [9,21,36]. Other reasons such as inclusion levels of dietary fiber, breed and BW of pig can also affect the results. All factors that affect the ATTD of OM can affect the ATTD of GE and therefore the DE value as the adaptation time increased. In our study, the DE value and the ATTD of nutrients except for ADF in the pigs adapted to diets for 7 d were greater than those adapted for 14, 21, or 28 d. On d 7, the gastrointestinal tract was not well adapted, and more nutrients may be used for bacteria activities, with less excretion to feces.

In our current study, the adaptation time had no effect on the ATTD of CP in the diet, which agreed with a previous study [8], but conflict with a report that the apparent digestibility coefficient of CP in a starch or NSP diet fed to sows for 2 to 5 weeks was greater than that in diets fed to sows only for 1 week [10]. This difference may be due to the different BW of pig, fiber sources and inclusion levels, which would result in different endogenous losses of nitrogen and different synthesis of microbial proteins in the large intestine as the adaptation time increased [8]. The adaptation time had no effect on the ATTD of ADF. Therefore, the main reason for the decreased ATTD of GE as well as decreased DE value is the decreased ATTD of NDF and OM in diet.

### Effects of adaptation time and inclusion level on the digestible energy value and the apparent total tract digestibility of gross energy and crude protein in sugar beet pulp

The ATTD of GE and DE value linearly decreased in the diet, but linearly increased in the ingredient SBP as the adaptation time increased from 7 to 28 d. This difference may be due to the fiber in SBP having greater digestibility compared with the fiber from the corn and SBM in the basal diet, and more short-chain fatty acids were absorbed as the adaptation time increased. The linearly increased ATTD of CP in SBP may be due to the longer adaptation time leading to more CP being used to synthesize protein for bacterial proliferation in the large intestine of pigs, which resulted in less excretion of CP in feces [37]. The possible reasons for the decreased ATTD of CP with increased SBP level may be because the high fiber in SBP increases the specific endogenous losses of AA [18,37]. Inclusion level had no effect on the ATTD of GE and DE value of SBP, however, the average value of DE in SBP determined in this study was less than that value [11]. The possible reasons for this difference may be due to the different variety of beet used for production of SBP, processing of SBP, and different animals used for evaluating the SBP.

The lower DE value in SBP compared with the corn grain [11] may be mainly due to a combination effect of the high fiber and low EE content in SBP.

### Ileal amino acid digestibility of sugar beet pulp

The AID and SID values of AA in corn grain were in good agreement with previous values [11] except that 90.42% for the AID and 91.61% for the SID of Arg were observed, respectively. The AID of all AA but Lys, Met, and Ala in SBP were greater than the values from NRC [11]. The SID of all AA but Ile, Leu, Lys, Met, and Ala in SBP were greater than previously reported values [11,38]. Even though containing comparable CP content to the corn grain, the lower AID or SID of CP and all tested AA in SBP compared with the corn grain indicated that SBP had a poor quality of CP and AA for pigs.

## CONCLUSION

The results of this study indicated that the ATTD of energy and nutrients were decreased as inclusion rate of SBP increased.

## Figures and Tables

**Table 1:** Analyzed composition of corn and sugar beet pulp (%, as-fed basis)1)

Item	Corn	Sugar beet pulp
Dry matter	89.0	90.5
Ether extract	3.3	0.8
Ash	1.3	7.3
Organic matter	87.7	83.2
Total dietary fibre	10.2	58.7
Soluble dietary fibre	0.5	23.9
Insoluble dietary fibre	9.7	34.8
Neutral detergent fibre	10.1	36.6
Acid detergent fiber	3.1	22.2
Gross energy (kcal/kg)	3,943	3,508
Crude protein	8.4	8.5
Indispensable amino acid		
Arg	0.37	0.31
His	0.24	0.22
Ile	0.25	0.29
Leu	1.01	0.54
Lys	0.24	0.45
Met	0.16	0.07
Phe	0.38	0.36
Thr	0.29	0.34
Trp	0.06	0.08
Val	0.41	0.48
Dispensable amino acid		
Ala	0.45	0.51
Asp	0.53	0.63
Cys	0.19	0.07
Glu	1.37	0.94
Gly	0.29	0.34
Pro	0.71	0.46
Ser	0.36	0.37
Tyr	0.27	0.36

1) Data are the mean of duplicate analyses of each ingredient.

**Table 2:** Ingredient and analyzed composition of experimental diets in Exp. 1 (%, as-fed basis)1)

Items	Inclusion level of sugar beet pulp (%)					

	0.0	14.6	24.4	34.2	43.9	53.7
Ingredient						
Corn	76.6	65.1	57.4	49.7	42.1	34.4
Soybean meal, 48% CP	21.0	17.9	15.8	13.7	11.6	9.5
Sugar beet pulp	-	14.6	24.4	34.2	43.9	53.7
Limestone	1.0	1.0	1.0	1.0	1.0	1.0
Dicalcium phosphate	0.6	0.6	0.6	0.6	0.6	0.6
Salt	0.3	0.3	0.3	0.3	0.3	0.3
Vitamin-mineral premix2)	0.5	0.5	0.5	0.5	0.5	0.5
Analyzed chemical composition						
DM	88.4	87.8	87.8	88.2	88.6	88.8
CP	16.8	15.1	14.3	13.6	12.6	12.2
Ash	4.0	4.7	5.3	5.5	6.1	6.4
OM	84.4	83.1	82.5	82.7	82.5	82.4
NDF	10.1	13.0	18.4	19.8	23.7	26.4
ADF	3.5	5.8	8.8	10.1	11.7	13.5
GE (kcal/kg)	3,867	3,804	3,757	3,694	3,706	3,694

1) All data are the results of a chemical analyses conducted in duplicate. DM, dry matter; CP, crude protein; OM, organic matter; NDF, neutral detergent fibre; ADF, acid detergent fibre; GE, gross energy.

2) Provided the following quantities per kg of complete diet: Mn, 50 mg (MnO); Fe, 125 mg (FeSO_4_·H_2_O); Zn, 125 mg (ZnO); Cu, 150 mg (CuSO_4_·5H_2_O); I, 50 mg (CaI_2_); Se, 0.30 mg (Na_2_SeO_3_); retinyl acetate, 4,500 IU; cholecalciferol, 1,350 IU; DL-α-tocopheryl acetate, 13.5 mg; menadione sodium bisulphite complex, 2.7 mg; niacin, 18 mg; vitamin B_12_, 27.6 μg; thiamine, 0.6 mg; pyridoxine, 0.9 mg; riboflavin, 1.8 mg; D-Ca-pantothenate, 10.8 mg; nicotinic acid.

**Table 3:** The ingredient and analyzed composition of the experimental diets in Exp. 2 (%, as-fed)

Items	Diets		

	Corn	Sugar beet pulp	N-free
Ingredient			
Corn	96.6	-	-
Sugar beet pulp	-	30.0	-
Corn starch	-	50.0	73.35
Soybean oil	-	2.0	3.0
Sucrose	-	15.0	15.0
Acetate cellulose1)	-	-	4.0
Dicalcium phosphate	1.7	1.5	2.5
Limestone	0.6	0.4	0.5
Sodium chloride	0.3	0.3	0.45
Chromic oxide	0.3	0.3	0.3
Potassium carbonate	-	-	0.3
Magnesium oxide	-	-	0.1
Vitamin-mineral premix2)	0.5	0.5	0.5
Analyzed composition			
Crude protein	8.03	2.54	0.35
Indispensable amino acid			
Arg	0.35	0.08	0.01
His	0.23	0.06	0.01
Ile	0.24	0.08	0.01
Leu	0.96	0.15	0.05
Lys	0.24	0.12	0.01
Met	0.14	0.01	0.00
Phe	0.33	0.11	0.03
Thr	0.25	0.09	0.01
Trp	0.05	0.01	0.00
Val	0.37	0.14	0.01
Dispensable amino acid			
Ala	0.42	0.15	0.02
Asp	0.49	0.18	0.02
Cys	0.16	0.01	0.11
Glu	1.21	0.28	0.03
Gly	0.26	0.10	0.01
Pro	0.63	0.14	0.02
Ser	0.34	0.11	0.01
Tyr	0.26	0.10	0.02

1) Made by Chemical Reagents Company (Beijing, China).

2) Provided the following quantities per kg of complete diet: Mn, 50 mg (MnO); Fe, 125 mg (FeSO_4_·H_2_O); Zn, 125 mg (ZnO); Cu, 150 mg (CuSO_4_·5H_2_O); I, 50 mg (CaI_2_); Se, 0.30 mg (Na_2_SeO_3_); retinyl acetate, 4,500 IU; cholecalciferol, 1,350 IU; DL-α-tocopheryl acetate, 13.5 mg; menadione sodium bisulphite complex, 2.7 mg; niacin, 18 mg; vitamin B_12_, 27.6 μg; thiamine, 0.6 mg; pyridoxine, 0.9 mg; riboflavin, 1.8 mg; D-Ca-pantothenate, 10.8 mg; nicotinic acid.

**Table 4:** The effect of adaptation time and inclusion level of sugar beet pulp on digestible energy (DE, kcal/kg, as-fed basis) value and nutrient digestibility (%) of experimental diets fed to pigs in Exp. 11)

Items	DE	GE	CP	DM	Ash	OM	NDF	ADF
Adaptation time								
7 d	3,295	87.73	84.64	87.92	53.52	90.15	74.11	72.15
14 d	3,259	86.80	83.91	86.83	48.38	89.33	73.09	73.09
21 d	3,262	86.82	83.74	86.71	46.68	89.30	71.25	71.65
28 d	3,264	86.93	84.20	86.81	46.98	89.41	73.45	72.93
Standard error mean	10.19	0.24	0.25	0.90	0.21	0.79	0.88	0.39
p-linear	0.008	0.009	0.377	<0.001	<0.001	0.005	0.143	0.812
p-quadratic	0.067	0.068	0.199	0.026	0.007	0.039	0.011	0.840
Inclusion level								
0%	3,529	91.28	92.15	91.25	55.27	92.95	67.74	65.80
14.6%	3,396	89.27	88.52	89.22	53.48	91.24	69.48	69.53
24.4%	3,286	87.47	85.57	87.43	50.73	89.79	73.27	73.95
34.2%	3,183	86.12	82.83	86.16	46.25	88.83	72.87	73.89
43.9%	3,152	85.02	80.09	84.98	45.55	87.89	76.59	75.23
53.7%	3,075	83.27	75.57	83.35	42.06	86.58	77.91	76.33
Standard error mean	14.21	0.38	0.40	1.26	0.36	1.26	1.36	0.57
p-linear	<0.001	<0.001	<0.001	<0.001	<0.001	<0.001	<0.001	<0.001
p-quadratic	0.009	0.913	0.058	0.827	0.499	0.740	0.809	0.121
Interactive effect								
p-day	<0.001	<0.001	<0.001	<0.001	<0.001	<0.001	<0.001	<0.001
p-level	0.06	0.067	0.508	0.004	<0.001	0.039	0.074	0.521
p-d×l	0.329	0.341	0.515	0.318	0.725	0.233	0.498	0.248

1) Values were least squares mean of six observations per treatment. DE, digestible energy; GE, gross energy; CP, crude protein; DM, dry matter; OM, organic matter; NDF, neutral detergent fibre; ADF, acid detergent fibre.

**Table 5:** The effect of adaptation time and inclusion level of sugar beet pulp on the digestible energy (DE, kcal/kg, as-fed basis) value, ATTD (%) of GE and CP of sugar beet pulp fed to pigs in Exp. 11)

Items	DE	GE	CP
Adaptation time			
7 d	2,927	75.56	65.88
14 d	2,901	74.86	70.84
21 d	3,022	77.99	73.57
28 d	3,025	78.08	74.45
Standard error mean	41.04	1.06	1.47
p-linear	0.026	0.026	<0.001
p-quadratic	0.754	0.754	0.266
Inclusion level			
14.6%	2,982	76.95	79.16
24.4%	2,889	74.55	72.53
34.2%	2,869	74.08	70.31
43.9%	3,042	78.54	69.02
53.7%	3,061	78.98	64.92
Standard error mean	55.13	1.39	1.74
p-linear	0.097	0.097	<0.001
p-quadratic	0.060	0.060	0.385
Interactive effect			
p-day	0.069	0.069	<0.001
p-level	0.006	0.006	0.001
p-d×l	0.023	0.023	0.024

1) Values were least squares mean of six observations per treatment. DE, digestible energy; ATTD, apparent total tract digestibility; GE, gross energy; CP, crude protein.

**Table 6:** The apparent ileal digestibility (AID, %) and standardized ileal digestibility (SID, %) of amino acids in corn and sugar beet pulp in Exp. 2

Item	AID		SEM^1)^	p-value	SID		SEM	p-value
	
	Corn	Sugar beet pulp			Corn	Sugar beet pulp		
Crude protein	78.47	39.20	2.12	<0.001	82.39	50.00	1.21	<0.001
Indispensable amino acid								
Arg	90.42	68.00	0.78	<0.001	91.61	69.26	0.77	<0.001
His	79.93	54.31	1.34	<0.001	82.68	57.30	1.34	<0.001
Ile	71.22	43.88	1.30	<0.001	75.59	50.23	1.16	<0.001
Leu	78.15	48.65	2.14	<0.001	81.51	52.53	2.02	<0.001
Lys	61.96	33.35	2.07	<0.001	65.45	37.03	2.09	<0.001
Met	74.19	48.54	1.67	<0.001	76.65	51.62	1.58	<0.001
Phe	79.59	56.74	1.27	<0.001	84.29	59.98	1.17	<0.001
Thr	64.46	32.51	1.50	<0.001	71.57	40.68	1.20	<0.001
Trp	75.25	41.07	0.86	<0.001	79.90	46.22	0.88	<0.001
Val	64.32	39.95	2.42	<0.001	69.19	48.92	2.58	<0.001
Dispensable amino acid								
Ala	65.66	35.21	1.78	<0.001	71.81	43.28	1.73	<0.001
Asp	77.16	49.93	1.87	<0.001	80.24	53.90	1.20	<0.001
Cys	73.32	45.77	1.71	<0.001	79.45	53.75	1.29	<0.001
Glu	84.48	48.49	1.35	<0.001	85.96	60.60	1.33	<0.001
Gly	63.75	36.39	2.27	<0.001	72.70	46.51	1.49	<0.001
Pro	64.37	41.50	2.00	<0.001	77.55	54.08	2.27	<0.001
Ser	72.92	43.88	1.46	<0.001	77.43	47.68	1.39	<0.001
Tyr	74.37	54.89	3.76	<0.001	83.98	64.01	4.64	<0.001

SEM, standard error mean.
